# Attention to Monocular Images Bias Binocular Rivalry

**DOI:** 10.3389/fnsys.2019.00012

**Published:** 2019-03-18

**Authors:** Manuel Moreno-Sánchez, J. Antonio Aznar-Casanova, Fernando Valle-Inclán

**Affiliations:** ^1^Department of Cognitive Processes, Universitat de Barcelona, Barcelona, Spain; ^2^Department of Psychology, Universidad de La Coruña, La Coruña, Spain

**Keywords:** visual awareness, visual attention, binocular rivalry, endogenous attention, attention

## Abstract

When monocular images cannot be fused, perception alternates between the two (or more) possible images. This phenomenon, binocular rivalry (BR), is driven by the physical properties of the stimuli (size, contrast, spatial frequency, etc.) but it can also be modulated by attention to features of one of the rival stimuli (Chong et al., 2005; Dieter et al., 2016) and by attentional demands independent of the BR assessment (Paffen et al., 2008). Instead of the perceptually demanding tasks previously used to bias BR, we designed a simple counting task. We monocularly presented a number of trials (around 10 min) with a set of symbols and asked participants to count them. We found that after this task, dominance durations decreased for the unattended channel, and did not change for the attended channel. The results parallel those of Paffen et al. (2008) and square nicely with Levelt’s second proposition, suggesting that the counting task effectively increased the sensibility of one channel which led to increased strength of the images presented to that channel. Alternatively, the results could be explained assuming that the non-attended channel was inhibited during the counting task, and the inhibition was carried over to the BR task.

## Introduction

When unfusable images are dichoptically presented, perception alternates between the possible percepts, which are usually two. This phenomenon, binocular rivalry (BR), is essentially stochastic and arises from competition at multiple levels in the visual system (Blake and Logothetis, 2002). The perceptual alternations in BR depend on the physical characteristics of stimuli (size, contrast, motion, etc.; Levelt, 1965), but are also under the influence of attentional processes. Helmholtz (1925) was the first to note that attending to one of the rival stimuli (i.e., counting the lines in one of the images) prolonged dominance durations for that stimulus. More recent research has shown that images immediately presented before the rivalry display tend to be the initial dominant image (Meng and Tong, 2004; Mitchell et al., 2004; Chong and Blake, 2006; Hancock and Andrews, 2007). Also, when observers have to track subtle changes in one of the rival stimuli, dominance durations increase for that image/channel (Chong et al., 2005; Hancock and Andrews, 2007; Dieter et al., 2015, 2016). Finally, when subjects attend to relevant stimuli mixed with irrelevant stimuli in a non-rivalry task, and then the relevant irrelevant stimuli are dichoptically presented, the dominance durations of the previously unattended stimulus decrease but the dominance of the attended stimulus does not change (Paffen et al., 2008).

In the experiments above, the biases in dominance duration were induced by tracking of changes in one of the rivalrous stimuli (Chong et al., 2005; Hancock and Andrews, 2007; Dieter et al., 2016), or after training on a binocular visual discrimination task (Paffen et al., 2008). It is not totally clear if less perceptually demanding tasks, performed outside the BR assessment task, would produce similar effects. We tested this idea using a simple monocular counting task and assessing BR before and after. The use of simple, easily discriminable and static to-be-attended stimuli provides a stricter test of the effects of endogenous attention on BR.

## Experiment 1

### Participants

All subjects gave written informed consent in accordance with the Declaration of Helsinki. All the experiments were approved by the local ethics committee (Bio-ethics committee of the University of Barcelona) and conducted in accordance with the Declaration of Helsinki of 1975 (as revised in Fortaleza, Brazil, October 2013). In all the experiments, participants had normal (or corrected to normal) visual acuity (20/20), and stereoacuity (at least 60 s arc, according to TNO test).

Fifty-two students (40 women) between 19 and 26 years (mean = 22.45; SD = 2.63), volunteered for the experiment. Two subjects did not show perceptual changes in some of the BR tasks, and were excluded. Participants were randomly assigned to left-attended (*N* = 16), right-attended (*N* = 19), and control groups (*N* = 15).

### Materials and Methods

#### Stimuli and Apparatus

The stimuli were presented on a 19-inch TFT screen (1,280 × 768 pixels). The rivalrous stimuli were anaglyphs with red and cyan square-wave gratings orthogonally oriented (±45°), with a spatial frequency of 0.82 cycle deg^−1^ and 70% contrast. They subtended a visual angle of 2.81 deg. The stimuli to be attended were sets of “Os” and “Xs.” The stimulus presentation and response collection were controlled with a C++ (Open-GL API) program running on a desktop with Windows 7.

#### Procedure and Data Analysis

The experimental procedure was the same in all experiments, except the attentional task. There were two BR sessions (each of them comprising four 1-min periods) in which the subjects pressed keys to indicate their perception. The rival display was a red and cyan anaglyph seen with the red filter on the left eye and the cyan filter on the right eye.

**Figure 1:**
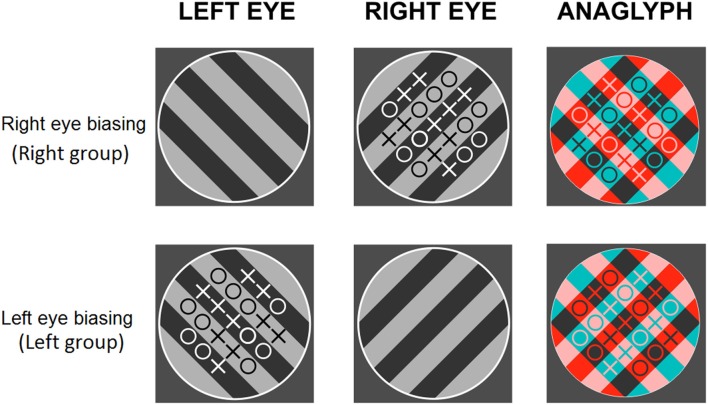
Experiment 1. Examples of the counting task: anaglyphs for the right- (upper row) and left- (lower row) attended conditions.

**Figure 2:**
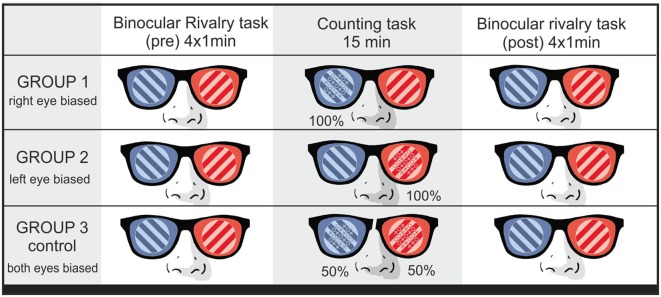
Experiment 1. Sequence of tasks for each group (row).

In between the two BR sessions, subjects saw the same rival targets with a set of O’s and X’s (21 in total) overlaid on one of them (see Figure 1). The difference between the two items varied randomly from trial to trial. Subjects were instructed to count those elements and indicate which one was more numerous by pressing a key. Following their response, a blank screen was presented for 1 s and the next display appeared. Subjects performed this task for around 10 min. In different groups, the symbols were presented to the left eye, right eye, or alternate between the eyes in different trials (control group). Figure 2 summarizes the procedure.

### Results

In the counting task, there were 101.37 trials per subject (SD = 6.59) with a duration of 8.19 s/trial, (SD = 1.24). Mean accuracy was 0.96.

**Figure 3:**
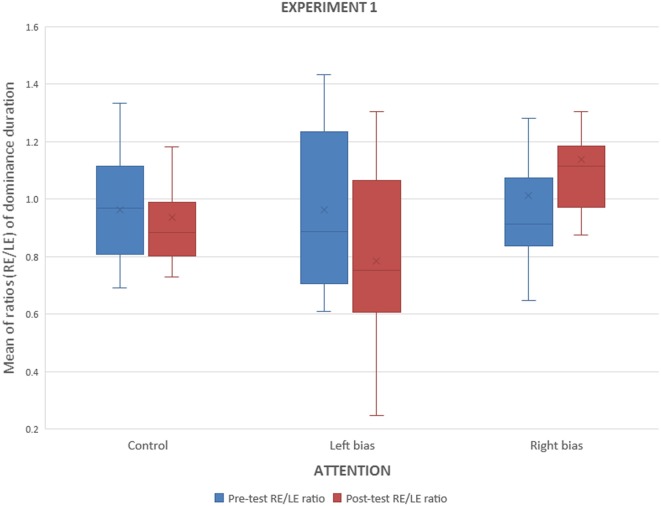
Experiment 1. Interocular ratios (right/left) of median dominance durations before and after the monocular counting task.

The interocular ratio (right eye/left eye) of dominance durations were calculated for the pre and the post BR tasks. Figure 3 shows that after performing the monocular counting task, the interocular ratio decreased for the left-attended group and increased for the right-attended. An ANOVA with factors attention (control, left attended, right attended) and time (before, after the BR task) showed an interaction of attention and time for dominance durations (*F*_(2,49)_ = 5.90, *p* < 0.005) and for alternations (*F*_(2,49)_ = 4.32, *p* < 0.02).

**Figure 4:**
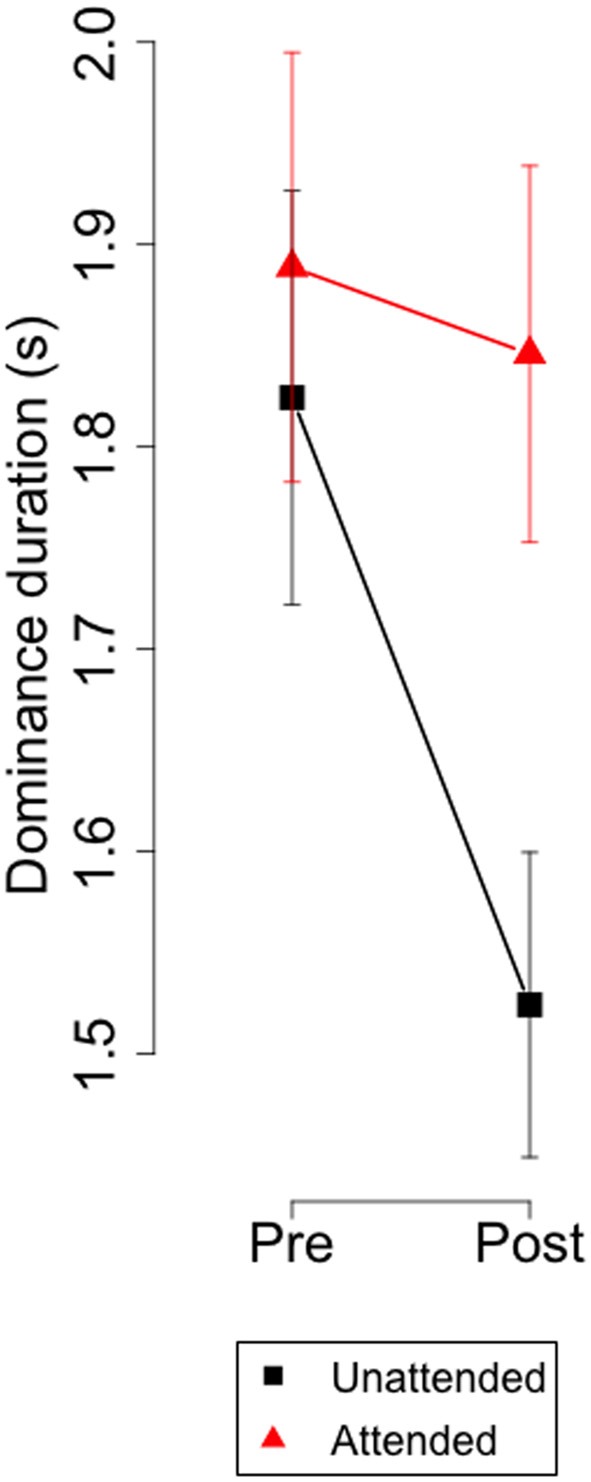
Experiment 1. Group means and 95% confidence intervals of dominance durations for the attended and unattended channel before and after the monocular attentional task.

The biases shown in Figure 3 could result from increases in attended eye, from decrements in the unattended eye, or a combination of both. Figure 4 depicts the dominance durations for the attended and unattended channels during the monocular attentional task. It shows that, after paying attention to a monocular set of symbols, dominance duration on the unattended eye decreased but did not change on the attended eye. These observations were confirmed with an ANOVA with factors: attention (attended, not attended) and time (before, after the attentional task) which revealed a significant interaction between attention and time (*F*_(1,35)_ = 9.53, *p* < 0.004).

### Discussion

The experiment showed that minutes later after paying attention to monocular sets of O’s and X’s overlaid on a rivalrous display, the dominance durations decreased on the unattended channel and did not change on the attended channel. This result differs from Chong et al. (2005), Hancock and Andrews (2007), and Dieter et al. (2016) who found that the dominance duration of the attended image increased while it did not change or decreased for the unattended stimulus. The main difference with our procedure is that these experiments demanded attention to some feature of the rivalrous display that was under continuous and subtle change. It seems plausible that perceptual load was much higher in those experiments than in ours, and if this were the critical difference, the results suggest that mild perceptual loads inhibit the unattended channel, while higher loads enhance neural responses in the attended channel (Zhang et al., 2012).

Our findings dovetail with those of Paffen et al. (2008) who trained subjects in a binocular motion discrimination task with relevant and irrelevant directions. The relevant and irrelevant stimuli (random dots) were used on a BR task before and after the perpetual discrimination task. They found the same pattern described here: previously not attended stimuli became less dominant, while the attended stimuli were unaffected. A straight forward interpretation of these findings would be that the irrelevant stimuli were suppressed in the presence of relevant stimuli (Paffen et al., 2008). On our experiment, the same logic would state that, in order to perform the counting task, the unattended channel and/or the background image, were inhibited.

Alternatively, it is tentative to suggest that monocular attention increased the effective contrast for the images presented to that channel as suggested by Chong and Blake (2006). This interpretation squares nicely with Levelt’s proposition 2 (Levelt, 1965) which predicts that increasing the contrast of a stimulus will decrease the dominance of the other stimulus and will not affect the dominance of the stimulus with increased contrast. The generality of Levelt’s propositions has been limited by recent research (see Brascamp et al., 2015, for a review) and, in particular proposition two does not seem hold when contrast of one stimulus is fixed at a low level and the other stimulus is varied over higher contrast levels (Brascamp et al., 2006; Moreno-Bote et al., 2010). These findings led to reformulate Levelt’s proposition as follows: changes in contrast of one eye affect the mean dominance duration of the highest contrast eye. According to this modified second proposition, we should expect longer dominances on the attended than on the unattended. However, the violations of Levelt’s second proposition have been generally found with smaller stimuli than the confirmations (Kang, 2009), and the size of our stimuli (2.8°) were in the range where confirmations of Levelt’s 2nd propositions have been reported.

There is no way we can decide whether monocular attention enhanced neural responses and boosted the contrast in the attended channel, or simply inhibited the unattended channel. However, there is a previous question we can explore. Were the effects due to functional changes in the monocular channels, independently of the images presented, or were they due to stimulus adaptation. To test this idea, the counting task in Experiment 2 presented the same grating and sets of O’s and X’s to both eyes, and Experiment 3 used different orientations in the attentional and in the BR tasks.

## Experiment 2

The experiment was designed to test whether indirect attention to an image, in a task not involving rivalry nor monocular selection, could replicate Experiment 1. It has been found that the initial dominance phase is affected by images shortly presented before the rivalry display (Mitchell et al., 2004; Chong and Blake, 2006; Hancock and Andrews, 2007), but we are not aware of studies showing similar effects on dominance durations.

### Method

#### Participants

Forty-five volunteers, students of Psychology (38 women) between 19 and 26 years (mean = 22.36 years; SD = 2.51 years), were randomly assigned to one of these groups: left-attended (*N* = 16), right-attended (*N* = 16), and control (*N* = 13).

#### Stimuli, Procedure, and Data Analysis

**Figure 5:**
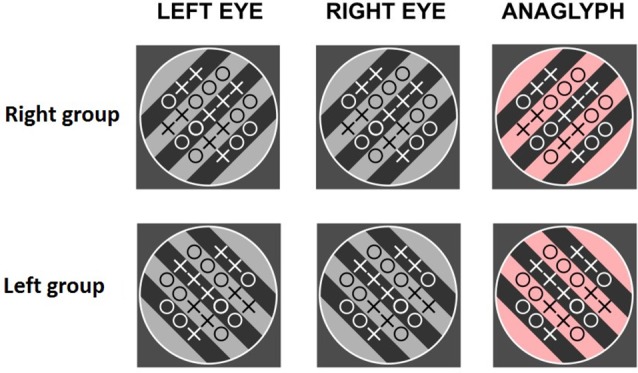
Experiment 2. Examples of the counting task. Different subjects saw right-, left-oriented gratings or both types of gratings, on different trials (control group).

The experiment was identical to Experiment 1, except that the counting task was binocular and the gratings had the same orientation on both eyes. Different groups of subjects saw left-oriented gratings, right-oriented gratings, or both on different trials (control group). Figure 5 illustrates these conditions. The data treatment and statistical analyzes were identical to those in Experiment 1.

### Results and Discussion

**Figure 6:**
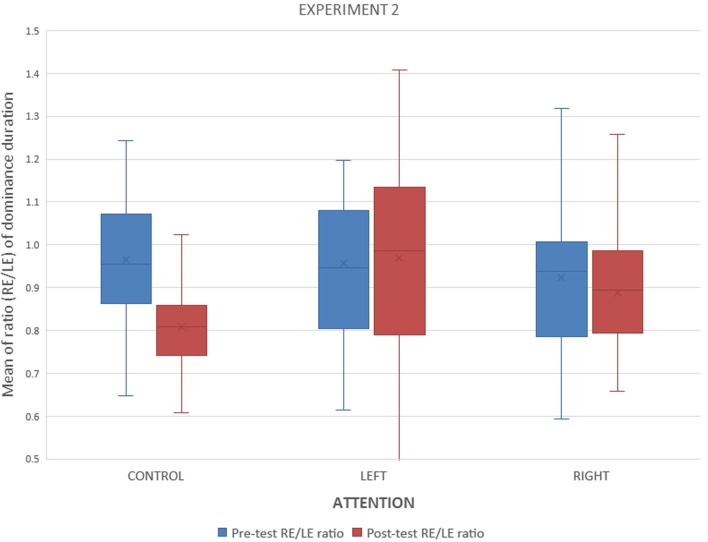
Experiment 2. Interocular ratios (right/left) of dominance durations before and after the monocular counting task for each group on the counting task.

In the counting task, there were 129.56 trials per subject (SD = 6.35) with a duration of 6.24 s per trial, (SD = 1.16). Mean accuracy was 0.94. Figure 6 summarizes the results obtained after calculating the interocular ratios (see Experiment 1) and indicates that the attentional task had no influence. The ANOVA confirmed this observation for the dominance durations (*F*_(2,42)_ = 2.351; *p* < 0.1).

Although it is difficult to argue based on null effects, these results suggest that indirect attention to an image minutes before the BR task, does not affect dominance durations neither alternations. It seems reasonable to assume that the presence of BR and the attention to a monocular image, or channel, are requisites to induce the biases in found in Experiment 1.

## Experiment 3

Knowing that mere exposure to a stimulus does not affect BR, we tried to dissociate the effects of the background image (the grating) from those of the attention within a monocular channel. Remember that in Experiment 1 the gratings were the same during the counting and BR tasks. Changing the rivalrous displays during the counting and the BR tasks should not affect the findings of Experiment 1 if the critical factor is the monocular presentation of the to-be-attended stimuli. In addition, we tried to control the possible cross-talks between the color filters using two groups of subjects, each with a different location of the cyan and red filters.

### Method

#### Participants

Eighty-four volunteers (70 women), between 19 and 26 years (mean = 22.36 years; SD = 2.51 years) participated in the experiment. Forty-four of them performed the experiment with the red and cyan filters over the left and right eyes, respectively. For the other 40 subjects, the location of the filtered was reversed. Participants were randomly assigned to left attend, right attend or attend both, on different trials, conditions during the counting task.

#### Stimuli, Procedure, and Data Analysis

**Figure 7:**
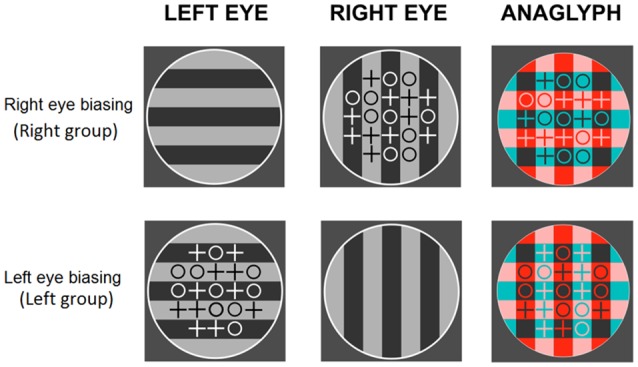
Experiment 3. Examples of stimuli during the counting task. Anaglyphs for the right- (upper row) and left-attended (lower row) groups. The control group saw both type of displays in different trials.

The only differences with Experiment 1 were the orientation of gratings (0 and 90°) during the counting task (see Figure 7) and the addition of a second group with the locations of the color filters reversed. Since preliminary analyzes showed no differences related to the location of the filters, the two groups were collapsed.

### Results and Discussion

In the counting task, there were 105.57 trials per subject (SD = 7.08) with a duration of 7.79 s/trial, (SD = 1.10). Mean accuracy was 0.94.

**Figure 8:**
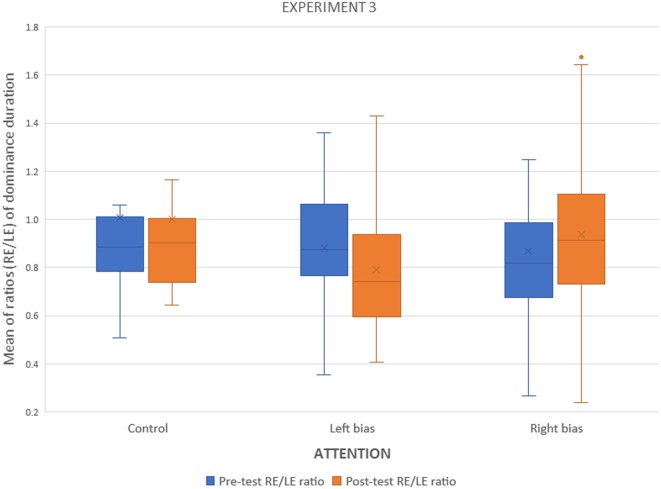
Experiment 3. Interocular ratios (right/left) of dominance durations before and after the monocular counting task.

The interocular ratios (right eye/left eye) for dominance and alternations were analyzed as in Experiment 1. The only significant effect for the alternation rate was an increase in the number of alternations in the second BR tasks (*F*_(1,83)_ = 43.59, *p* < 0.0001). Figure 8 depicts dominance results, which show the pattern seen in Experiment 1, confirmed by an interaction of attention condition × time × eye (*F*_(2,83)_ = 5.27, *p* < 0.007).

**Figure 9:**
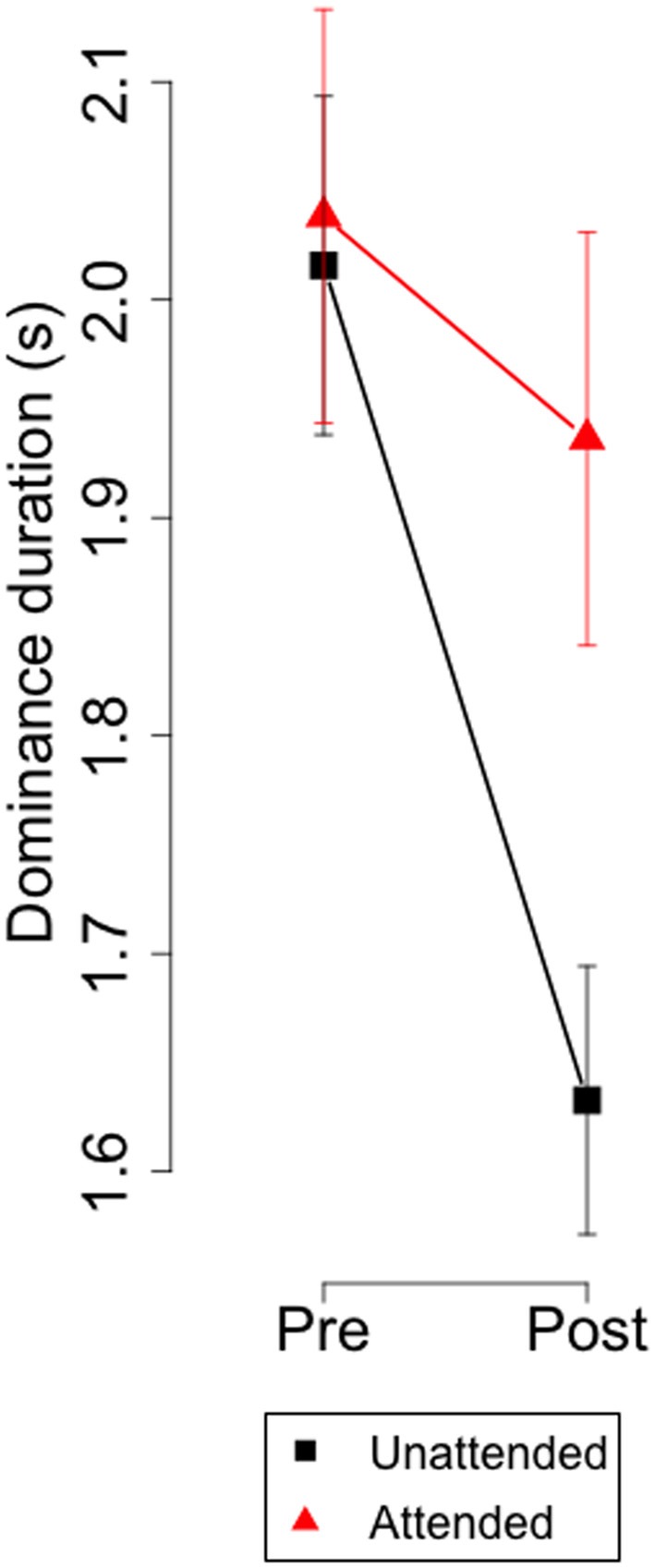
Experiment 3. Group means and 95% confidence intervals of dominance durations for attended and unattended channel before and after the monocular attentional task.

Again, to elucidate whether these biases were caused by increments in the attended channel, decrement in the unattended, or a combination of both, a second analysis including only the left and right attended groups, resulted in a strong interaction between attention condition and time (*F*_(1,57)_ = 10.83, *p* < 0.002; Figure 9).

The results for dominance duration replicate the main finding of Experiment 1, namely, the decreased dominance durations for the unattended monocular channel. Since the gratings were different in the attentional and the BR tasks, the conclusion is that attention to a monocular channel is the critical variable. However, the size of the effect in Experiment 3 was slightly smaller than in Experiment 1, making it reasonable to think that adaptation to the grating played some role in Experiment 1. We analyzed both experiments together looking for some effect related to the experiment, and there were none.

The main difference with Experiment 1 was the absence of effects related to attention on the alternation rate, which lead us to consider the previously reported finding as unreliable, since the sample size was double in this experiment.

## General Discussion

After some minutes seeing short-term (8 s, as an average) rivalry displays with monocular elements to be attended, dominance was reduced for the non-attended monocular channel, without changes in the attended channel. Although the task required attention, it is possible that the results just reflect the differences between the two images. It is known that stimuli with higher density contour tend to dominate (Levelt, 1965) and subjects would have learned to use one of the monocular channels when confronted with rival displays. Within this interpretation, the experiments are closer to a low-level perceptual learning than to an endogenous attentional effect. However, were this the case, the attended channel should show longer dominances after the counting task, in overt contradiction with the results.

Discarding the low-level perceptual learning leads to interpretations related to endogenous attention. A plausible interpretation would be that the non-attended channel was inhibited during the counting task, and this effect was carried over to the BR task (as Paffen et al., 2008; Vergeer et al., 2016, have proposed in different experimental setups). Alternatively, the counting task could have enhanced neural responses in one channel and as a consequence, the stimuli presented to that channel would be have higher contrast than their contralateral partners. Contrary to common sense, increasing the contrast of one stimulus does not affect dominance for that stimulus, instead, it reduces dominance of the opposite stimulus. This is the second proposition of Levelt (1965) which is valid for relatively large stimuli (Kang, 2009) and for smaller stimuli within a range of contrasts (Moreno-Bote et al., 2010; Brascamp et al., 2015).

In summary, we found that monocular attention prior to a BR task, reduces the dominance in the non-attended channel, without changes in the attended channel. It should be noted that the attentional effects here reported last for minutes (as in Paffen et al., 2008). These results are consistent with either or both interpretations. First, the neural response in the attended channel was enhanced, which is equivalent to increase the contrast of the stimuli presented to that channel. Second, monocular attention suppressed the neural responses on the unattended channel.

## Author Contributions

MM-S, JA-C and FV-I designed the experiment, conducted the research, analyzed the data and wrote the manuscript. All authors contributed to the manuscript.

## Conflict of Interest Statement

The authors declare that the research was conducted in the absence of any commercial or financial relationships that could be construed as a potential conflict of interest.
